# Inventory and Analysis of Controlled Trials of Mobile Phone Applications Targeting Substance Use Disorders: A Systematic Review

**DOI:** 10.3389/fpsyt.2021.622394

**Published:** 2021-02-22

**Authors:** Rubaab Bahadoor, Jean-Marc Alexandre, Lucie Fournet, Thibaut Gellé, Fuschia Serre, Marc Auriacombe

**Affiliations:** ^1^University of Bordeaux, Bordeaux, France; ^2^Addiction Team Phenomenology and Determinants of Appetitive Behaviors, Sanpsy CNRS USR 3413, Bordeaux, France; ^3^Pôle Addictologie et Filière Régionale, CH Charles Perrens and CHU de Bordeaux, Bordeaux, France; ^4^Department of Psychiatry, Center for Studies of Addiction, Perelman School of Medicine, University of Pennsylvania, Philadelphia, PA, United States

**Keywords:** substance use disorder, mHealth, efficacy, mobile applications, systematic review

## Abstract

**Background:** Less than 20% of people with addictions have access to adequate treatment. Mobile health could improve access to care. No systematic review evaluates effectiveness of mobile health applications for addiction.

**Objectives:** First aim was to describe controlled trials evaluating the effectiveness of smartphone applications targeting substance use disorders and addictive behaviors. Secondly, we aimed to understand how the application produced changes in behavior and craving management.

**Method:** A systematic review based on PRISMA recommendations was conducted on MEDLINE, CENTRAL, and PsycINFO. Studies had to be controlled trials concerning addictive disorders (substance/behavior), mobile application-based interventions, assessing effectiveness or impact of those applications upon use, published after 2008. Relevant information was systematically screened for synthesis. Quality and risk of bias were evaluated with JADAD score.

**Results:** Search strategy retrieved 22 articles (2014-2019) corresponding to 22 applications targeting tobacco, alcohol, other substances and binge eating disorder. Control groups had access to usual treatments or a placebo-application or no treatment. Eight applications showed reduced use. Most of the applications informed about risks of use and suggestions for monitoring use. Twelve applications managed craving.

**Discussion:** Heterogeneity limited study comparisons. Duration of studies was too short to predict sustainable results. A reduction of craving seemed related to a reduction in use.

**Conclusion:** There is a lack of robust and comparable studies on mHealth applications for addiction treatment. Such applications could become significant contributors in clinical practice in the future so longer-termed double-blind studies are needed. Targeting craving to prevent relapse should be systematic.

## Introduction

Substance use disorder (SUD) and behavioral addiction are a major public health concern affecting 10–15% of the world population (1). Regarding legal and illegal substance use, worldwide prevalence has remained globally stable over the last few years. Even though European countries have the highest prevalence of alcohol and tobacco consumption, a decrease has been observed over the past decade (2, 3). However, global health burden remains substantial. Beyond significant costs in health care, SUD human cost is alarming: more than 7 million deaths per year for tobacco, more than 3 million for alcohol and 450,000 for other substances (3–5).

Addiction is a chronic disorder which persists beyond abstinence. More than withdrawal symptoms, craving is considered to be a major contributor to repeated relapses (6–9). Craving is listed as one of the core diagnostic criteria in the DSM 5, placing it as a main symptom of addiction and a legitimate target for treatment (10). Despite addiction being a severe condition, it is estimated that overall less than 20% of people with an addictive disorder have access to adequate treatment and this is true across countries (4, 11, 12).

Mobile health (mHealth) may help to reduce this “treatment gap” by improving early diagnosis and access to treatment (11, 12). It has been defined by the World Health Organization as any medical intervention based on mobile devices (13). With the dissemination of mobile services in developed and developing countries, patients with chronic diseases are particularly concerned worldwide. These technologies represent a considerable opportunity to access people in need of medical help, where and when it can be difficult in practice. mHealth is a means to overcome social and territorial disparities in health (14, 15). The digitalization of healthcare services has improved access to information, professional support and medical assistance due to its asynchronous means of communication that abolishes barriers such as traveling time and costs and schedule conflicts with healthcare professionals (16, 17).

mHealth can also contribute to raise awareness among young substance users who consider themselves in good health, while this population is very much affected by risky behaviors (18–20). In France, only 12% of teenagers have never experienced tobacco, alcohol or cannabis; revealing a high accessibility of these substances (21) and this is true also in Europe, North America, and Australia (22–24). Smartphones, as an autonomous tool, may be a promising new direction to improve the commitment of young adults to care and improving self-efficacy and empowerment (25).

In clinical practice, health applications may offer a complementary approach to usual direct contact care. The combined application of cognitive behavioral therapy (CBT) with smartphone applications could increase access to effective interventions (26). Immediate intervention through these applications can enhance therapeutic effectiveness, consolidate and maintain behavioral change on a long-term basis (27), thus, helping the patient to be independent without being isolated from professional support.

While more than 300,000 health applications currently exist (28), including hundreds of “cessation support” applications, few of them have been clinically tested before being put on the market (17, 29). The majority of these applications have no proof of validity (30–32). Some applications even encourage substance use, implicitly or explicitly (29, 30, 33). Yet, studies suggest that mobile applications can positively influence our health behavior (34, 35). Some web-based or instant messaging interventions have demonstrated short- to mid-term effectiveness in reducing use (36, 37). To date, no systematic review has evaluated the effectiveness of mobile applications for the treatment of addictive disorders (32, 33, 38). The aim of this literature review was to identify and describe controlled treatment trials on mobile health applications which support behavior change among users with problematic behaviors or substance uses by the reduction of use or abstinence. Secondly, we aimed to understand how the application produced changes in use or behavior and the management of craving.

## Methods

### Review Protocol

This systematic literature review was based on the ≪ Preferred Reporting Items for Systematic Reviews and Meta-analyses ≫ (PRISMA) recommendations (39).

### Information Source and Search

Keywords were defined around 4 criteria (mobile applications, use disorder, effectiveness, excluding web-based interventions) and linked by Boolean operators to generate the following MESH equation: ((smartphone app^*^OR mobile app^*^)) AND ((substance use disorder OR addict^*^ OR addictive behavior OR cessation OR recovery OR craving OR alcohol OR smok^*^ OR tobacco OR cannabis OR marijuana OR heroin OR cocaine OR opioid OR gambl^*^ *OR binge eating OR porn)) AND ((efficacy OR effectiveness OR impact OR validity)) NOT ((web-based intervention OR Internet))*.

Literature searches were performed using MEDLINE, Cochrane central register of controlled trial (CENTRAL) and PsycINFO, up to 1st July 2019 (Table 1).

**Table 1:** MESH terms used for database searching.

**a.Mobile applications** 1) Smartphone app* 2) Mobile app* b. Addiction/craving 3) Substance use disorder 4) Addict* 5) Addictive behavior 6) Cessation 7) recovery 8) Craving 9) Alcohol 10) Smok* 11) Tobacco 12) Cannabis 13) Marijuana 14) Heroin	15) Cocaine 16) Opioid 17) Gambl* 18) Binge eating 19) Porn **c. Effectiveness** 20) Efficacy 21) Effectiveness 22) Impact 23) Validity **d. Web-based interventions** 24) Web-based intervention 25) Internet


### Eligibility Criteria and Study Selection

Studies were included if they met the following criteria: controlled trials concerning addictive disorders (substance/behavior), mobile application-based interventions, assessing the effectiveness or impact of those applications upon use. Addictive behaviors already added in DSM-5 (pathological gambling) or to be considered for further revisions because of important clinical data and research progress [binge eating, pornography (40)] were considered in this review. The research was limited to English and French articles that were published after 2008 which corresponds to the year of first release of health applications.

Age, sex, or nationality of the sample population were not included in the selection criteria. Articles doing a descriptive review of mobile applications, study protocols with no results on efficacy, and literature reviews were excluded.

The articles were first screened by their title and abstract. If relevant, full-text articles were read entirely for a second level selection. Database access and reference management were done by Endnote X6 software.

### Data Collection Process and Synthesis

Each selected paper was screened for relevant information such as whether the application treated one or more addictions, its functionalities, the target population, the randomization, the characteristics of the control group(s) and the results on use and/or craving. The quality of the study and risk of bias was evaluated by the JADAD score (41); a good methodological quality was defined by a score above 3/5.

## Results

### Study Selection

**Figure 1:**
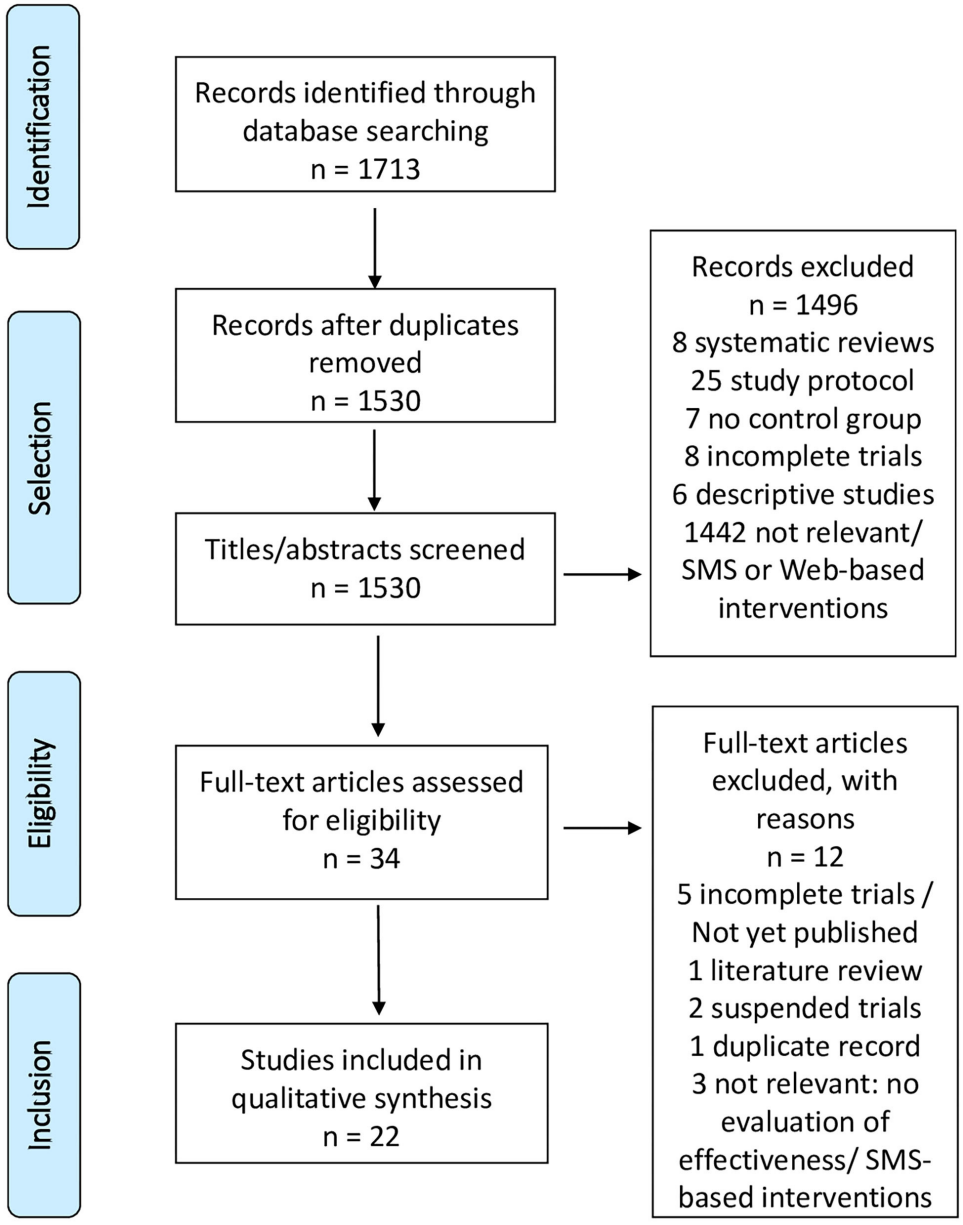
PRISMA flowchart.

The initial search found 1,713 articles. After screening by titles and abstracts, 34 articles were retained and thoroughly reviewed. Twenty-two controlled studies met our eligibility criteria. The selection steps are shown in Figure 1. The 22 selected articles concerned 22 applications (Table 2) focused on: tobacco (12 articles) (25, 42–52), alcohol (8 articles) (53–57, 59, 60), other substances (1 article) (61), and binge eating disorder (BED) (1 article) (62). The “A-CHESS” and “SmartQuit” (version 1.0 and 2.1) applications were each studied by two different teams (44, 56). One study evaluated two alcohol cessation applications (“Promillekoll” and “PartyPlanner”) (58). No application was dedicated to multiple addictions.

**Table 2:** List of applications identified by the systematic review.

**Tobacco**
***Crush the Crave* (25)** ***Quit Advisor Plus* (42)** ***SmartQuit*** *(version 1.0 et 2.1)* **43, 44)** ***SmokeFree* (45)** ***SmokeBeat* (46)** ***Craving to Quit* (47)** ***PhoS* (48)** ***Coach2Quit* (49)** ***Stop-Tabac* (50)** ***Can't Even Quit* (51)** ***SmokeFree Baby* (52)**
**Alcohol**
***Alcooquizz* (53)** ***Drink Less* (54)** ***A-CHESS* (55, 56)** ***TeleCoach* (57)** ***Promillekoll* (58)** ***Party Planner* (58)** ***LBMI-A* (59)** ***Ray's Night Out* (60)**
**Other substances**
***S-Health* (61)**
**Binge eating disorder**
***Noom Monitor* (62)**

The majority of studies were published since 2017 (*n* = 18) and a minority (*n* = 4) in 2014 and 2015, no studies were published before. The detailed analysis of each study is presented in Tables 3–5. A total of 39,031 participants were included in the studies (tobacco: 34,174; alcohol: 4,716; other substances than alcohol and tobacco: 75; BED: 66) whose duration ranged from 1 to 12 months (average 5 months). The studies were conducted in 11 different countries (Table 3). Two studies included participants aged 16 and over (51, 60) and the other studies included adults.

**Table 3:** Characteristics of controlled trials (ranged by addictive disorder).

**AuthorsDate and countries**	**Duration of study**	**Baseline characteristics of participants**	**Method**	**Principal results(Reduction of consumption and/or abstinence)**	**Impact on craving**
**TOBACCO**					
**Baskerville et al**. 2018 Canada (25)	6 months	*N* = 1,599 *Characteristics of participants:* daily smokers, 19–29 years, 73% low nicotine dependence (HSI score)	RCT, superiority trial, 2 parallel groups, ITT analysis, Crush the crave (CTC) (*n* = 820) v/s placebo app (OnRQ) (*n* = 779), self-reported continuous abstinence	*Abstinence*: 6-month follow-up: 7.8% for CTC v/s 9.2% for OnRQ; OR 0.83, 95% CI 0.59-1.18, *p* = 0.30 30-day point prevalence abstinence: 14.4% for CTC v/s 16.9% for OnRQ (OR 0.82, 95% CI 0.63–1.08) *Reduction of consumption:* Smoking less than a pack per day: Baseline: 25.7% CTC v/s 25.7% OnRQ (*p* = 0.99) 6-month follow-up: 23.8% CTC v/s 24.4% OnRQ (*p* = 0.86)	Not evaluated
**BinDhim et al**. 2018 USA Australia Singapore United Kingdom (42)	6 months	*N=* 684 *Characteristics of participants:* daily smokers, ≥ 18 years. Fagerström < 4 (49.6%) = 5 (13.7%) > 6–10 (36.7%)	RCT, parallel group, Quit Advisor Plus (intervention) v/s placebo app (informative), self-reported continuous abstinence	*Abstinence*: 1-month follow-up: 28.5% for intervention app v/s 16.9% for informative app (RR 1.68; 95% CI 1.25–2.28). Effect sustained at 3 months (23.8 vs 10.2%; RR 2.08; 95% CI 1.38–3.18) and 6 months (10.2 vs. 4.8%; RR 2.02; 95% CI 1.08–3.81). *Reduction of consumption*: (not specified)	Not evaluated
**Bricker et al**. 2014 USA (43)	2 months	*N=* 196 *Characteristics of participants:* ≥ 18 years, ≥ 5 cigarettes (cig) per day, not using other smoking cessation interventions. HSI: SmartQuit: 4.9 (SD: 2.5) QuitGuide: 4.7 (SD: 2.4)	RCT, pilot study, observational, SmartQuit (*n* = 98) v/s QuitGuide (*n* = 98), self-reported continuous abstinence	*Abstinence*: 2-month follow-up: 13% for SmartQuit v/s 8% for QuitGuide (OR = 2.7; 95% CI 0.8–10.3, *p* = 0.123) *Reduction of consumption*: (not specified)	Increase in craving acceptance for SmartQuit group (*p* < 0.04) only. High acceptance of craving strongly associated to abstinence at 2-month follow up (OR 6.1; 95% CI 3.0–15.2)
**Crane D, Ubhi HK, Brown J, West R**. 2018 United Kingdom (45)	3 months	*N =* 28 112 *Characteristics of participants:* daily or occasional smokers, ≥ 18 years, low to high nicotine dependence, at least 1 use of app	RCT, 1:1 parallel group, per-protocol analysis, intensive version of app (14 228) v/s minimal version (13 884), self-reported abstinence	*Abstinence:* 3-month follow-up: 19.3% for intervention group (*n* = 234/1213) v/s 13.8% for control group (*n* = 124/901) (OR: 1.50, 95% CI = 1.18–1.91, *p* < 0.001) *Reduction of consumption*: (not specified)	Not evaluated
**Dar R**. 2018 Israel (46)	1 month	*N =* 40 *Characteristics of participants:* 18-45 years, ≥ 5 cig per day Fagerström: -Intervention group: (M: 12.50, SD 3.32) -Control group: (M:19.95, SD 8.56)	RCT, pilot study, intervention group (*n* = 20) v/s control group (‘wait list') (*n* = 20), self-reported number of cig per day	*Abstinence*: (not specified) *Reduction of consumption:* Significant decline in smoking rate at 30 day-trial (*p* < 0.001) for intervention group only	Not evaluated
**Garrison et al**. 2020 USA (47)	6 months	*N =* 505 *Characteristics of participants:* 18–65 years, (mean) 16 cig per day, ≤ 3 months abstinence the previous year	RCT, ITT, parallel group: MMT-ES (*n =* 245) v/s ES (*n =* 260), survey at 1, 3, and 6 months. Smokerlyzer breath CO monitor. Measurement of craving by CEQ.	*Abstinence:* 6-month follow-up: (mean) quit rate 11.1%, no significant difference between the groups (MMT-ES, 9.8%; ES, 12.1%; χ^2^(1) = 0.43, *p* = 0.51). *Reduction of consumption:* At 6 months: significant decrease in number of cig per day (*p* < 0.0001), of craving intensity (*p* < 0.0001), of craving frequency (*p* < 0.0001), and increase in mindfulness (*p* < 0.05), similar in both groups	Association between craving and smoking reduced in the MMT-ES group throughout the trial v/s ES.
**Hassandra et al**. 2017 Finland (48)	6 months	*N =* 44 *Characteristics of participants:* 18–65 years, >10 cig per day, no psychiatric comorbidity or other addictions	RCT, pilot trial, 3 weeks behavioral counseling program on benefits of physical activities, additional aid to quit, 3–7 days after quit date: training on relapse prevention and craving management. Intervention group (*n* = 25) (app + BCT) v/s control group (*n* = 19) (BCT only), self-reported abstinence	*Abstinence*: No significant decrease in quit rate between the two groups (36% intervention group (*n* = 16/44) v/s 32% control group (*n* = 14/44)) *Reduction of consumption:* (not specified)	No significant decrease in number of relapse or craving.
**Krishnan N**, **Elf JL, Chon S, Golub JE**. 2018 USA (49)	1 month	*N =* 102 *Characteristics of participants:* daily smokers, ≥18 years. Nicotine dependence not specified	RCT, pilot trial, 1:1, brief intervention (BI) + CO-monitoring+ Coach2Quit v/s BI only, Biochemical verification of carbon monoxide (CO) level (breath and urine test) of cessation	*Abstinence*: At 1 month: 1 participant quit in each arm *Reduction of consumption:* CO level and number of cig per day similar in both groups [(intervention: −3.0 [interquartile range (IQR) −12.0, 2.0] control: −2.5 [IQR −9.0, 2.0]) et (intervention: −5.5 [IQR −14.0, −1.0]; control: −6.0 [IQR −10.0, −2.0]), respectively].	Not evaluated
**Mavrot C**, **Wittwer S, Etter JF**. 2017 Switzerland (50)	6 months	*N =* 2,892 *Characteristics of participants:* daily or ex- smokers, ≥18 years, (mean) 16 cig per day, 15 min after waking up	RCT, ITT, 1:1 parallel group, intervention group (*n =* 1,449) v/s control group (*n =* 1,443) (placebo app), self-reported number of cig per day, time before first cig	*Abstinence*: At 3 months: 16.2% intervention group v/s 15.7% control group, OR = 1.04; 95% CI [0.85–1.28], *p* = 0.685. At 6 months: 11.9% intervention group v/s 12.2% control group, OR = 0.97 CI [0.77–1.22], *p* = 0.819. Quit rate among ex-smokers similar in both arms at baseline. *Reduction of consumption:* (not specified)	Not evaluated
**O'Connor M, Whelan R, Bricker J, McHugh L**. 2019 Ireland (44)	6 months	*N =* 150 *Characteristics of participants:* ≥18 years, ≥10 cig per day since at least 1 year, no medication Fagerström: 4.7	RCT, ITT, 3-arm parallel groups: Combined group (Smartquit + “ACT”) (*n* = 50) v/s “ACT” group (*n* = 50) v/s BCT group (*n* = 50). 6 weeks and 90 min sessions of “ACT” and BCT Biochemical verification of abstinence at 6 weeks and 6 months	*Abstinence*: At 6 weeks: 36% combined group v/s 20% “ACT” group v/s 24% BCT group (*p* > 0.05) At 6 months: 24% combined group v/s 24% “ACT” group v/s 20% BCT group (*p* > 0.05) *Reduction of consumption:* Significant decrease for “SmartQuit” group at 6 weeks compared to “ACT” group (*p* = 0.017) and combined group (*p* = 0.013). Not significant at 6 months (*p* = 0.930 and *p* = 0.759 respectively)	Not evaluated
**Peiris et al**. 2019 Australia (51)	6 months	*N =* 49*Characteristics of participants:* aboriginal smokers > 16 yearsNicotine dependence not specified	RCT, pilot trial, 1:1, intervention group (app) (*n* = 25) v/s control group (usual treatment) (*n* = 24), carbon monoxide breath test	*Abstinence*:At 6 months: 4.5% (*n* = 1/22) intervention group v/s 0% (*n* = 0/24) control group.*Reduction of consumption:* (not specified)	Not evaluated
**Tombor et al**. 2018 United Kingdom (52)	1 month	*N =* 565*Characteristics of participants:* pregnant smokers, >18 years, smoke at least once per weekMedium nicotine dependence (HSI: 2.5)	RCT, factorial design (2x2x2x2x2), evaluation of 5 modules in each arm: intervention group (complete version) v/s control group (minimal version), self-reported number of smoke-free days	*Abstinence*: (not specified)*Reduction of consumption:*No significant effect of the 5 modules on use	Not evaluated
**ALCOHOL**					
**Bertholet N, Godinho A,Cunningham JA**. 2018 Canada Switzerland (53)	6 months	*N =* 977*Characteristics of participants:* ≥18ans, AUDIT≥8, ≥15 drinks per weekAUDIT: (M: 18.3, SD: 7.1)	RCT, multicentric, 1:1 parallel group, intervention group (*n* = 461) v/s control group (*n* = 516), simple blind, ITT analysis, self-reported number of drinks	*Abstinence:* (not specified)*Reduction of consumption:*Number of drinks per week:Baseline: 28.9 (SD: 16.7) in both arms6-month follow-up: 18.9 (SD: 15.0) intervention group v/s 21.4 (SD: 18.0) control group (IRR 0.93, 95% CI 0.84–1.03, *p* = 0.17)No significant decrease on number of drinks on one occasion (IRR 0.99, 95% CI 0.93–1.06, *p* = 0.81)	Not evaluated
**Crane D, Garnett C, Michie S, West R, Brown J**. 2018 United Kingdom (54)	2 months	*N = 672Characteristics of participants:* excessive drinkers, ≥18years,AUDIT (M: 19.1, SD: 6.56) willing to quit	RCT, randomized block design, full version (*n* = 336) v/s minimal version(*n* = 336) of app, ITT, self-reported number of drinks per week and AUDIT score	*Abstinence:* (not specified)*Reduction of consumption:*Non-significant, but numerically larger reduction in number of drinks per week and AUDIT score full version at 2-month follow-up	Not evaluated
**Gajecki M, Andersson C, Rosendahl I, Sinadinovic K, Fredriksson M, Berman AH**. 2017 Sweden (57)	3 months	*N =* 186*Characteristics of participants:* University students with excessive consumption, AUDIT (M: 10.7, SD: 3.9), quantity: 9.3 drinks/week	RCT, 3-arm study: intervention group (*n* = 93) v/s “wait list” group (access to app after 6 weeks) (*n* = 93), compared to control group of another trial (58), assessment by DDQ and AUDIT	*Abstinence:* (not specified)*Reduction of consumption:*Decrease of excessive alcohol consumption in intervention group (45.3%) v/s “wait list” group (50%) v/s control group (72.7%) at 6 weeks, not maintained at 12 weeks Decrease in quantity of drinks for intervention group only (−4.76, 95% CI [−6.67,−2.85], Z = −2.09, *p* = 0.037), at 6 weeks, not maintained at 12 weeks Decrease in drinking frequency (−0.83, 95% CI [−1.14, −0.52], Z = −2.04, *p* = 0.041) at 6 and 12 weeks.	Not evaluated
**Gajecki M**, **Berman AH, Sinadinovic K, Rosendahl I, Andersson C**. 2014 Sweden (58)	7 weeks	*N =* 1,932 *Characteristics of participants:* University students, hazardous drinking, AUDIT: (M: 14,08, SD: 5,00), quantity: 17 drinks/week, frequency: 3,5/week, binge drinking: 1,87/week.	RCT, 3-arm study, 1:1:1, per-protocol analysis, Promillekoll group (*n* = 643) v/s PartyPlanner group (*n* = 640) v/s control group(*n* = 649), assessment by DDQ, AUDIT and eBAC	*Abstinence:* (not specified) *Reduction of consumption:* No impact on consumption in any of the groups. Per-protocol analysis show an increase in drinking frequency for Promillekoll app compared to control group (*p* = 0.001)	Not evaluated
**Glass et al**. 2017 USA (55)	12 months	*N =* 349 *Characteristics of participants:* Patients ≥18 years, alcohol use disorder (DSM 4), discharge from 5 residential treatment programs	RCT, cause and effect study, recruitment 2 weeks before discharge, outpatient addiction treatment + A-CHESS v/s mutual help + A-CHESS, access to app during 8 months, surveys by telephone at 4, 8, and 12 months, self-reported consumption	*Abstinence:* An increase in abstinence was observed in both group with no mediation effect of A-CHESS *Reduction of consumption:* A decrease in the number of risky drinking days was observed in both groups. A positive correlation with A-CHESS was found in the outpatient addiction treatment group. The A-CHESS group had increased odds of obtaining outpatient addiction treatment (OR = 2.14; 95% IC [1.27–3.61]).	Not evaluated
**Gonzalez VM**, **Dulin PL**. 2015 USA (59)	6 weeks	*N =*54 *Characteristics of participants:* 18–45 years, alcohol use disorder (DSM5) SADQ: LBMI-A: (M:13,82, SD 6,51)>> low severity Placebo app + bibliography: (M: 16,50, SD: 6,52)>> medium severity	Controlled pilot trial, not randomized, “LBMI-A” (*n* = 28) v/s placebo app + bibliography (*n* = 26), self-reported consumption	*Abstinence:* LBMI-A group produced significant increase in quit rate (*p* < 0.001) whereas the control group did not (*p* = 0.324) *Reduction of consumption:* LBMI-A group showed significantly greater decrease in number of drinks per week (*p* = 0.003) as well as binge drinking (*p* = 0.007) than placebo app.	Not evaluated
**Gustafson DH, McTavish FM, Chih M et al**. 2014 USA (56)	12 months	*N =* 349 *Characteristics of participants:* Patients ≥18ans, alcohol use disorder (DSM 4), enrolled in 5 residential programs (BCT, motivational intervention, psychoeducation), no psychiatric comorbidities	RCT, parallel group 1:1, randomized block design, intervention group (usual treatment + A-CHESS) (*n* = 170) v/s control group (usual treatment only) (*n* = 179), self-reported consumption	*Abstinence:* Significant increase in quit rate at follow-up compared to control group (51.9 v/s 39.6%; *p* = 0.03) *Reduction of consumption:* Signification decrease in number of days of risky drinking in intervention group compared to control group (M = 1.39 days v/s 2.75 days respectively; *p* = 0.003; 95% CI [0.46–2.27]).	Not evaluated
**Hides et al**. 2018 Australia (60)	6 months	*N* = 197 *Characteristics of participants:* young adults, 16–25 years, hazardous drinking, > 4 drinks/ occasion during the last month, AUDIT > 7 (45.1%)	RCT, immediate access to app v/s differed access (1 month), assessment by AUDIT score	*Abstinence:* (not specified) *Reduction of consumption:* Increase in knowledge at 1 month without consequence on use (*p* < 0.001, d = 0.46) Decrease in quantity of consumption at 6 months, mainly among men with problematic used	Not evaluated
**OTHER SUBSTANCES THAN ALCOHOL AND TOBACCO**					
**Liang D, Han H, Du J, Zhao M, Hser YI**. 2018 China (61)	1 month	*N =* 75 *Characteristics of participants:* Adult, use of heroin or other substances during the last month, methadone maintenance treatment	RCT, S-Health (*n* = 50) v/s control group (SMS only) (*n* = 25), self-reported consumption and urine test* *morphine, methamphetamine, ketamine, marijuana	26.2 v/s 50% of positive urine test in the intervention and control group respectively (*p* = 0.06) Decrease in number of days of consumption the previous week: intervention group (M = 0.71, SD = 1.87) v/s control group (M = 2.20, SD = 3.06) (*p* < 0.05)	Not evaluated
**BINGE EATING DISORDER**					
**Hildebrandt et al**. 2017 USA (62)	6 months	*N =* 66 *Characteristics of participants:* ≥ 18 years, diagnosis of bulimia nervosa or BED, DSM 5 or DMS IV with once weekly binge eating and/or purging	RCT, ITT, Noom monitor + BCT (self-help guide) v/s BCT only, assessment by EDE-Q	No significant decrease in eating disorder behavior at 6 months. Decrease in compensatory behavior similar in both groups at follow-up	Not evaluated

*ACT, acceptance and commitment therapy; app, application; AUDIT, alcohol use disorders test; BCT, behavioral cognitive therapy; CEQ, craving experience questionnaire; CI, confidence interval; cig, cigarettes; DDQ, daily drinking questionnaire; eBAC, estimated blood alcohol concentration; EDE-Q; eating disorder examination questionnaire; ES, experience sampling; HSI, heaviness smoking index; IRR, incidence rate ratio; ITT, intention-to-treat; M, mean; MMT-ES, mindfulness training- experience sampling; OR, odd ratio; RCT, randomized controlled trial; RR, relative risk; SD, standard deviation*.

**Table 4:** Characteristics of interventional and control groups (ranged by addictive disorder).

**Authors**	**Name of application** **Functionalities/specificities**	**Control group(s)Functionalities**
**TOBACCO**		
**Baskerville et al**. (25)	*App:* Crush the Crave (CTC) version 2.1 *Functionalities*: - Information: relapse, craving, medications - Set quit date and help to choose the quit mode, - Track money saved, health benefits and number of cig per day *Specificities:* - Positive reinforcement: motivational message, virtual rewards - Identify cues and help to manage craving (web-based distractions)	On the Road to Quiting (OnRQ)- Self-help guide, - Informative, - Similar contents to CTC
**BinDhim NF, McGeechan K, Trevena L**. (42)	*App:* Quit Advisor Plus *Functionalities*: - Information: quitting options (benefits/risks) - Quitting agenda - Quitting benefits tracker *Specificities:* - Decision-aid app to help choose quit method - Mandatory information - Customized, systematic and daily motivational messages	Placebo app: - Non-mandatory information on quit methods - No structured information on benefits/harms of different quit methods
**Bricker et al**. (43)	*App:* SmartQuit version 1.0 *Functionalities:* - Information on medications - Motivational messages on reasons to quit - Customized quit plan (treatments, social support) - Tips on craving managements *Specificities:* - Craving management based on acceptance and commitment therapy. - Track number of craving passed without smoking	QuitGuide app: - Based on US guidelines - Information on medications - Motivates to quit - Personalized quit plan - Craving management strategies
**Crane D, Ubhi HK, Brown J, West R**. (45)	*App:* SmokeFree *Functionalities:* - Information on benefits of quitting - Track progress: number of days without smoking, savings *Specificities:* - Daily missions on craving prevention and management, based on behavioral cognitive therapy. - Virtual rewards	Placebo app: - Minimal version - Excluded missions
**Dar R**. (46)	*App:* SmokeBeat *Functionalities:* - Monitoring: detected hand to mouth movements predicting smoking, number of cig per day *Specificities:* (“smart Watch” or connected bracelets) - Real-time identification of smoking	“Wait list” group: - No intervention
**Garrison et al**. (47)	*App:* Craving to quit *Specificities:* - Mindfulness training - Meditation techniques - Conscience of craving (RAIN: Recognize, Accept, Investigate and Note)	Placebo app: - Minimal version - “Experience sampling/ES”: monitoring of use and real-time craving
**Hassandra et al**. (48)	*App:* PhoS (Physical activity over smoking) *Functionalities:* - Information on risks of smoking - Motivational messages to quit *Specificities:* - Craving management by physical exercises	Usual treatment: - Behavioral cognitive therapy
**Krishnan N,Elf JL, Chon S, Golub JE**. (49)	*App:* Coach2Quit *Functionalities:* - Measurement of exhaled CO (eCO) twice per day (automatic reminder) - Information on consumption based on eCO	Usual treatment: - Brief intervention
**Mavrot C,Wittwer S, Etter JF**. (50)	*App:* Stop-Tabac *Functionalities:* - Customized tracker: consumption, savings, progress - Interactive coach: personalized messages on abstinence, craving, high risk situations - Help to manage craving: messages (SMS) and counseling on relapse prevention, relaxation exercises - Professional help through quitline/forum *Specificities:* - Customization - Systematic and automatic messages at degressive frequency (except for relapse)	Placebo app: - Consumption tracker - Few impersonalized messages.
**O'Connor M, Whelan R, Bricker J, McHugh L**. (44)	*App:* SmartQuit version 2.1 (2Morrow) *Functionalities:* - Personalized quit plan - Determine reasons to quit *Specificities:* - Based on acceptance and commitment therapy (ACT) - Track craving passed without smoking - Combined to face to face ACT	Control group 1: - Face to face ACT Control group 2- Behavioral cognitive therapy
**Peiris et al**. (51)	*App:* Can't Even Quit *Functionalities:* - Automated motivational messages (frequency established by user) on abstinence and relapse prevention while experiencing craving - Consumption tracker	Control group: - No intervention - Participants encouraged to use smoking cessation support
**Tombor et al**. (52)	*App:* SmokeFree Baby *Functionalities:* 5 modules: 1) Identity: persuasion and modulation of self-image (positive image, accepting his identity as ex-smoker) 2) Stress management: information on tobacco and stress, stress management techniques, relaxation exercises 3) Effects on health: information on harms of smoking and benefits to quit 4) Face to face: social support 5) Behavior: encourage behavior change, alternative solutions to smoking	Placebo app: - Few tips on cessation - Non-interactive
**ALCOHOL**		
**Bertholet N, Godinho A,Cunningham JA**. (53)	*App:* Alcooquizz *Functionalities:* - Information on use and its consequences - Monitoring of consumption - Tool to design a driver *Specificities:* - Not automated (app use depends on user) - Reinforcement of self-efficacy (fix personal objectives) - Customized normative feedback - eBAC calculation	Control group: - No intervention
**Crane D, Garnett C, Michie S, West R, Brown J**. (54)	*App:* Drink Less *Functionalities:* (5 interventional module) 1) Customized normative feedback 2) Cognitive bias retraining 3) Self-affirmation: identity change 4) Action planning 5) Self-monitoring of consumption and harms	Placebo application: - Minimal version - Informative - Not personalized
**Gajecki M, Andersson C, Rosendahl I, Sinadinovic K, Fredriksson M, Berman AH**. (57)	*App:* TeleCoach *Functionalities:* - Information on consequences of hazardous drinking - Monitoring of number of drinks/days *Specificities:* - Relapse prevention skills training: 1) ≪ Say No to Alcohol ≫: analysis of high risks situations, refusal exercise: “say no to alcohol” 2) ≪ Feel better without alcohol ≫: relaxation exercises, positive thinking, urge surfing training	Control group 1: - Access to app after 6 weeks Control group 2 (58): - No intervention
**Gajecki M,Berman AH, Sinadinovic K, Rosendahl I, Andersson C**. (58)	*App:* 1) Promillekoll 2) PartyPlanner *Functionalities:* Promillekoll: - Real-time monitoring of number of drinks and eBAC calculation - Specific strategies to maintain eBAC below 0.06% to prevent dangerous drinking - Information on use and eBAC PartyPlanner - Simulate and plan use before an event; compared to real consumption afterwards - Monitoring of number of drinking and eBAC calculation	Control group: - No intervention
**Glass et al**. (55)	*App:* A-CHESS *Functionalities:* - Information on addiction - Motivational messages - Monitoring of drinks - Interaction with other drinkers and experts - Testimony of patients and families *Specificities:* - Identify high risk situations (GPS) - Craving management by behavioral cognitive therapy - Customization - Combined to outpatient addiction treatment	Mutual help: - Associated to A-CHESS
**Gonzalez VM,Dulin PL**. (59)	*App:* LBMI-A *Functionalities:* - 7 psycho-educative modules *Specificities:* - Based on real-time assessment and intervention - Real-time monitoring of craving (intensity, cues) - Real-time solutions to manage craving	Control group: - Placebo app: Brief intervention during 1 h on internet on behavior change - + bibliography: documents on craving management, how to refuse drinking and overcome relapse
**Gustafson DH, McTavish FM, Chih M et al**. (56)	*App:* A-CHESS *Functionalities:* - Information on addiction - Motivational messages - Monitoring of drinks - Interaction with other drinkers and experts - Testimony of patients and families *Specificities:* - Identify high risk situations (geolocation) - Craving management by behavioral cognitive therapy - Customization	Usual treatment: - Medications - Behavioral cognitive therapy
**Hides et al**. (60)	*App:* Ray's Night Out *Functionalities:* - Information, motivation and technics to maintain consumption goals - Information about how to reduce risks of intoxication - Information on physical and behavioral consequences of intoxication - Monitoring of number of drinks per event	“wait list” group: - Differed access to app at 1 month
**OTHER SUBSTANCES**		
**Liang D, Han H, Du J, Zhao M, Hser YI**. (61)	*App:* S-Health *Functionalities:* - Information on risks reduction (text message at predefined time) - Customized motivational messages on positive/negative affects *Specificities:* - Daily surveys (predefined time/ upon request) on 1) craving intensity, 2) affects, 3) cues (emotions, place, context), 4) responses to stimuli, 5) social context (alone or with a particular person - Personalization	Placebo app: - Few informative messages
**BINGE EATING DISORDER**		
**Hildebrandt et al**. (62)	*App:* Noom monitor *Functionalities:* - Self-monitoring: exercise, meal/snacks, compensatory behaviors, craving, weight - Personal notes *Specificities:* - Combined use with behavioral cognitive therapy (self-help guide)	Control group: - Behavioral cognitive therapy only

*app, application; cig, cigarettes; eBAC, estimated blood alcohol concentration; US, United States (of America)*.

**Table 5:** Bias risk assessment- JADAD score (ranged by addictive disorder).

**Authors Application**	**Randomization?**	**Appropriate randomization?**	**Double blind?**	**Appropriate method of double-blinding?**	**Withdrawal/drop-outs described?**	**Total**
**TOBACCO**						
**Baskerville NB et al**. (Crush the Crave) (25)	Yes	Yes	Yes	Yes	Yes	5
**BinDhim NF, McGeechan K, Trevena L**. (Quit Advisor Plus) (42)	Yes	Yes	Yes	Yes	No	4
**Bricker et al**. (SmartQuit 1.0) (43)	Yes	Yes	Yes	No (not described)	Yes	3
**Crane D, Ubhi HK, Brown J, West R**. (SmokeFree) (45)	Yes	Yes	No	-	Yes	3
**Dar R**. (SmokeBeat) (46)	Yes	No	No	-	No	0
**Garrison KA et al**. (Craving to Quit) (47)	Yes	No (not described)	Yes	Yes	Yes	3
**Hassandra et al**. (PhoS) (48)	Yes	Yes	Yes	Yes	Yes	5
**Krishnan N,Elf JL, Chon S, Golub JE**. Coach2Quit) (49)	Yes	No (not described)	Yes	No (not described)	Yes	1
**Mavrot C,Wittwer S, Etter JF**. (Stop-Tabac) (50)	Yes	Yes	No	-	Yes	3
**O'Connor M, Whelan R, Bricker J, McHugh L**. (SmartQuit 2.1/2Morrow) (44)	Yes	Yes	No	-	Yes	3
**Peiris et al**. (Can't Even Quit) (51)	Yes	Yes	Yes	No (not described)	Yes	3
**Tombor et al**. (SmokeFree Baby) (52)	Yes	Yes	No	-	Yes	3
**ALCOHOL**						
**Bertholet N, Godinho A,Cunningham JA**. (Alcooquizz) (53)	Yes	Yes	No	-	No	2
**Crane D, Garnett C, Michie S, West R, Brown J**. (DrinkLess) (54)	Yes	Yes	No	-	No	2
**Gajecki M, Andersson C, Rosendahl I, Sinadinovic K, Fredriksson M, Berman AH**. (TeleCoach) (57)	Yes	Yes	No	-	Yes	3
**Gajecki M,Berman AH, Sinadinovic K, Rosendahl I, Andersson C**. (Promillekoll/PartyPlanner) (58)	Yes	Yes	No	-	Yes	3
**Glass et al**. (A-CHESS) (55)	Yes	Yes	No	-	Yes	3
**Gonzalez VM,Dulin PL**. (LBMI-A) (59)	No	-	No	-	Yes	1
**Gustafson DH, McTavish FM, Chih M et al**. (A-CHESS) (56)	Yes	Yes	No	-	Yes	3
**Hides et al**. (Ray's Night Out) (60)	Yes	Yes	No	-	Yes	3
**OTHER SUBSTANCES**						
**Liang D, Han H, Du J, Zhao M, Hser YI**. (S-Health) (61)	Yes	No	No	-	Yes	1
**BINGE EATING DISORDER**						
**Hildebrandt et al**. (Noom monitor) (62)	Yes	Yes	No	-	Yes	3

### Tobacco Addiction Applications

#### Characteristics of Studies

One study targeted users aged 19–29 years (25). One application was specifically intended to support pregnant women (52) and another one for aboriginal Australian population (51). The level of severity of addiction varied between studies. Two studies included people with high severity (Heaviness smoking index (HSI) > 5, Fagerström> 7) (43, 46) and two others, with medium severity (his = 3, Fagerström = 5) (44, 52).

One application was compared to a self-help guide with similar contents to the application (25), three applications were compared to group therapy or brief intervention (44, 48, 49) and two other control groups did not have access to any intervention (46, 51). Other studies compared the active application to a placebo version of the application, mainly for informational or monitoring purposes [(42, 43, 45, 47, 50, 52); Tables 3, 4].

#### Effectiveness of Applications

The evaluation criteria of the studies were self-reported abstinence (25, 42, 43, 45, 46, 48, 50, 52) or biologically verified abstinence (expired /urinary level of carbon monoxide (CO)) (44, 47, 49, 51) or self-reported reduction in use (46, 52).

The rate of abstinence for the “Quit Advisor Plus” application was at 28.5% at 1 month compared to 10.2% at 6-month follow-up. Nevertheless, the overall quit rate for “Quit Advisor Plus” and for the remaining 2,214 participants of “SmokeFree,” who had variable nicotine dependence levels, was significantly higher compared to the placebo application, at 6 and 3 months, respectively. The “SmokeBeat” application (46) showed a significant reduction, at 1 month, in the number of cigarettes per day, among adult smokers with severe addiction (Fagerström score: 12.50 and 19.95 for interventional group and control group, respectively). Five other applications did not show a sustained reduction in tobacco use (25, 44, 47, 49, 52). The other studies did not specify the reduction of use [(42, 43, 45, 48, 50, 51); Table 3].

#### Functionalities of Applications

The main functions of the applications were information on the risks of tobacco use, the benefits of abstinence, the different modes of cessation and monitoring of tobacco use, financial savings and health gains due to quitting. Some applications had special features, such as personalization of data (42–44, 50), the particular modes of detecting tobacco use (46, 49) or specific craving management techniques [(43–45, 47, 48); Table 4].

#### Impact of Interventions on Craving

Among the eight applications which managed craving (25, 43, 44, 47, 48, 50, 51, 54), three of them (43, 47, 48) evaluated the effectiveness of the intervention on this symptom. Unlike “PhoS” (48), “SmartQuit 1.0” (43) showed a positive impact on craving with a higher quit rate and “Craving to Quit” (47), a reduction in craving intensity as well as a decrease in the association between craving and tobacco use (Table 3).

#### Methodological Quality of Studies—Risk of Bias

Two studies had low methodological quality (JADAD score <3) (46, 49) due to a non-optimal randomization (46) or the lack of description of randomization and double-blinding (49). Five studies were single-blinded [(44–46, 50, 52); Table 5].

### Alcohol Addiction Applications

#### Characteristics of Studies

Three studies aimed University students (57, 58) or users aged 16–25 years (60) engaged in hazardous drinking [Alcohol Use Disorder Test (AUDIT) score >6 for women, >8 for men, >4 drinks per event] (57, 60). Other studies included users with alcohol use disorder (AUD) according to the DSM IV (55, 56) and 5 (59) criteria or AUDIT score >12 (53, 54). Two studies recruited patients with AUD who were discharged from residential treatment programs (55, 56).

Two applications were compared to the usual CBT program or brief intervention (55, 56, 59), and one application was compared to a placebo version which was non-customizable and for information purposes only (54). One control group had delayed access, at 1 month, to the application (60) and three others had no intervention [(55, 56, 59); Tables 3, 4].

#### Effectiveness of Applications

The main outcome was alcohol reduction in quantity and/or frequency, either self-reported (53–57, 59) or evaluated by the AUDIT score and/or the daily drinking questionnaire (DDQ) (54, 57, 58, 60). Complete alcohol cessation was sought in three studies (55, 56, 59).

Three studies, concerning the two applications “A-CHESS” (55, 56) and “LBMI-A” (59) found a significant reduction in use as well as an increase in abstinence, among patients who were diagnosed with AUD, at 12 months and 6 weeks, respectively. Using “A-CHES” application was also positively correlated with a better adherence to outpatient addiction treatment. A positive correlation between the use of “A-CHESS” and the decrease in risky drinking days was found (55). The “TeleCoach” (57) application showed a significant decrease in the frequency of use, without any impact on the quantity, at 3-month follow-up while “Ray's Night Out” (60) showed a reduction in the quantity of alcohol use only at 1 month assessment. The “Promillekoll” application that estimated the blood alcohol concentration (BAC), showed a higher frequency of alcohol use (58). Compared to the control group, no change in drinking behavior were reported in the other studies [(53, 54, 58); Table 3].

#### Functionalities of the Applications

Most of the applications informed about the consequences of risky drinking and monitored the number of drinks. Three applications estimated the BAC (53, 58). One application used cognitive bias retraining by alcohol eviction games to review the user's approach to alcohol use (54). Three applications prevented situations of high risk of relapse by identifying craving in real time (55–57, 59). The self-determination theory which aimed at developing competency, relatedness, and autonomy was used in one application [(55, 56); Table 4].

#### Impact of Interventions on Craving

Although three applications managed craving, the effectiveness on its reduction was not sought in the corresponding studies: “A-CHESS” (55, 56), “LBMI-A” (59), and “TeleCoach” [(57); Table 3].

#### Methodological Quality of Studies—Risk of Bias

Five studies were of good methodological quality (JADAD score > 3) (55–58, 60). One study was not randomized (59). None of the studies were double-blinded (Table 5).

### Other Substances Addiction Application

#### Characteristics of Study

Only one study concerning the “S-Health” application was identified (61). The participants were users of other substances (ex: heroin, amphetamines.) in methadone treatment for opioid addiction (Tables 3, 4).

The control group had access to a placebo version of the application that only provided information on use by instant messages.

#### Effectiveness of Application

The number of days of use was self-reported daily and a multi-drug urine test was performed weekly by a clinician. A significant decrease in the number of days of use was observed in the interventional group. However, the positivity of the urine test was not statistically different between the two groups (Table 3).

#### Functionalities of the Application

Multiple surveys were conducted daily, systematically or upon request, about the context and personal state in which craving occurred, its expression and subject's response. The application also informed about reduction of HIV risk behavior and provided educational materials by text messages (Table 4).

#### Impact of Interventions on Craving

Even if the application dealt with craving, the impact of the intervention on this symptom was not sought, only substance use was reported (Table 3).

#### Methodological Quality of Study—Risk of Bias

This study had a low methodological quality (JADAD score = 1). The method of randomization was incorrect as the distribution of participants between the two groups was uneven. The study was not conducted in double-blind condition (Table 5).

### Binge Eating Addiction Application

#### Characteristics of Study

Only one study concerning the “Noom monitor” application was found (62). Participants were eligible if they met the diagnostic criteria of bulimia nervosa or binge eating disorder according to the DSM 5 or DSM IV with once weekly binge eating and/or purging.

The combined use of the application to behavioral cognitive therapy (BCT) was compared to BCT alone (Tables 3, 4).

#### Effectiveness of Application

The change in eating disorder behavior, with or without compensatory behaviors was evaluated by the Eating Disorder Examination Questionnaire (EDE-Q). No significant difference was found in the decrease of binge eating episodes or compensatory behaviors in both arms (Table 3).

#### Functionalities of the Application

The application served as a self-monitoring tool to record activities (physical exercises, meals/snacks, compensatory behaviors, craving, weight, personal notes; Table 4).

#### Impact of Interventions on Craving

The effect of the intervention on craving was not evaluated in this study (Table 3).

#### Methodological Quality of Study—Risk of Bias

This double-blind study had a good methodological quality [JADAD score = 3; (Table 5)].

## Discussion

### Synthesis of the Main Results

The aim of this systematic review of the literature was to identify and describe published controlled trials concerning health applications which support addiction behavior change among problem users, to substance or behavior addictions. We identified 22 trials regarding 22 applications. Each application targeted a unique addiction: tobacco, alcohol, other substances, and binge eating. The results of this review suggest that very few of these applications have shown compelling evidences of their efficacy upon abstinence or reduction of use or craving.

### Critical Analysis of Effective Applications

A total of 8 applications reported results supporting effectiveness (3 for tobacco, 4 for alcohol, and 1 for other substances use). Among the smoking addiction applications, “Quit Advisor Plus” (42), “SmokeFree” (45), and “SmokeBeat” (46) showed significant change in use behavior compared to controls, at 6, 3, and 1 month, respectively. Even if the rate of abstinence was higher for “Quit Advisor Plus,” a constant decline in quit rates was observed throughout the study. The number of abstinent participants was, numerically, half as important at 6-month follow-up which implies that more than half of them relapsed (42). The two other studies had important attrition bias (45, 46). The retention rates were extremely low (7.5%) for “SmokeFree,” despite massive recruitment, leading to inequalities between the characteristics of the study arms (45). Disparities were also found for “SmokeBeat” (46), where the control group had a higher tobacco addiction level due to error in randomization, which could, partially, explain the lack of effectiveness of the intervention in this group. Both “SmokeFree” (45) and “SmokeBeat” (46) were conducted over a too short duration to predict sustainable results on efficacy. Furthermore, no sub-group analysis was made to determine which level of severity of addiction could be more receptive to this type of intervention.

For applications which treated AUD, “A-CHESS” was positively correlated with improvement of use behavior among diagnosed patients, during aftercare, over 12 months. Findings in both studies were consistent. A better compliance to treatment was found in the intervention group. “A-CHESS” was the only application whose study was conducted during aftercare which represents a crucial moment for therapeutic adherence (55, 56). The impact of this application could be explained by the mechanism of behavior change based on self-efficacy and the fact that patients were encouraged to be proactive in their care by seeking social support in critical moments. The long study duration supports the effects of the intervention on a long-time basis.

The efficacy of “LBMI-A” (59), “TeleCoach” (57), and “Ray's Night Out” (60) was less convincing. The poor methodological quality, the short study duration as well as the lack of statistical power for “LBMI-A” impaired the quality of the study and did not allow relevant conclusions to be drawn (59). As for “TeleCoach” (57), the exclusive decrease in the frequency of use has no clinical value. The reduction in the quantity of use for “Ray's Night Out” (60), only at 1 month, was attributed, by the authors, to an assessment effect where an unconscious change in habits of use might occur at inclusion. No plausible argument supports the effect of those applications on behavior change.

For other substances, “S-Health” tested on patients with opioid use disorders showed a positive impact on the reduction of use (61). However, biological verification did not correlate these results at 1-month assessment. This could be in part explained by the detection method and the duration of the study. Change in use behavior could also have been overestimated by memory bias due to retrospective self-report of use and randomization bias.

### Critical Analysis of Ineffective Applications

Fourteen applications [9 for tobacco (25, 43, 44, 47–52), 4 for alcohol (53, 54, 58), and 1 for BED (62)] were considered to be ineffective. However, “Crush the crave” targeting smoking, which did not show greater efficacy than the manual guide, had a high quit rate (230/1599). Also, 30 more participants smoked less than one pack per day while using the application (25). Several factors may have influenced these results such as the frequency of use (higher for the guide) or the on-demand solicitation of the application which may be perceived as a burden by the user. The clinical impact of these findings is substantial. Moreover, the mechanism of delivery being different, the mobile application can be more easily disseminated than a paper booklet.

The use of BAC in the 'Promillekoll' application showed an unexplained increase in alcohol use (58). The calculation of BAC so as to limit use, in various studies, has not been proven effective (53, 58, 60).

Several biases have been identified in the other trials making the results on efficacy difficult to interpret, such as confusion factors with the control groups (44, 48, 54, 62) or attrition bias (44, 46, 54) between the two arms, the short study duration (less than 6 months) (43, 45, 46, 49, 52, 54, 57–59, 61) or the low statistical power of some trials (43, 44, 46, 48, 51, 57, 59–62) which lead to an under-estimation of the results.

### Craving Management

Twelve applications managed craving. “A-CHESS,” which showed probative results on change in use, specifically monitored craving and proposed real-time solutions to manage this symptom (55, 56). Even if, “Craving to Quit” (47) and “SmartQuit” (43) did not show significant effect on use, their craving management techniques seemed effective, on the short-term, on the intensity of craving, the association of craving and use or a better acceptance of the symptom which was correlated to a higher prevalence of abstinence. The lack of impact of physical activities on craving for the “PhoS” application could be explained by the fact that both study arms received information on the benefits of physical activities prior to the test (confusion factor) (48).

The other applications did not systematically evaluate the impact of the interventions on craving which made it difficult to determine whether the craving management techniques, used in those applications, are really effective.

### Validity of Results

Two applications (out of 8) which were considered effective by the authors had poor methodological quality (46, 59) due to inappropriate randomization. The JADAD score, used in this review, had certain limits. Double-blinding was not always possible between the study groups due to heterogeneous comparators (e.g., application compared to a paper booklet or no intervention). Some information could have been omitted by authors due to volume restriction of journals. Applications which are considered effective in one population, in a particular country should be tested in other contexts before any conclusion can be made on generalizability.

### Prospects of Improvement for Applications

More randomized controlled trials (RCTs) are required over a sufficiently longer period, minimum 12 months, and on a larger scale to be able to predict sustainable results. Information and monitoring are important features of health applications, however, the active involvement of the user (e.g., by daily tasks) could be more effective in enhancing the effects of the intervention. Mobile health interventions should continue to target the psychological mechanisms implied in behavior change, such as self-efficacy. A more systematic consideration of craving by the applications should be considered. The lack of support for craving might explain the failure to maintain change in behavior observed for some applications (42, 54, 57). The impact of these interventions must be measured in different contexts (with or without treatment, on different severity of addiction, in various sociodemographic contexts) to better understand their limitations and the profile of patients who could be more receptive to this type of intervention.

## Limitations

Some limitations of this review are to be acknowledged. First, searches were led in only three databases. Every effort was made to ensure that this review of the literature was comprehensive and encompassed all available and relevant literature however. Articles published elsewhere will not have been considered, however, we searched comprehensive databases for articles with the best methodological qualities. Articles not published in English or French language would have been missed by the search methodology. Secondly, selected applications are recent and further studies on their impact are in progress. Thirteen trials concerning addiction recovery applications have been identified in the Clinical Trial database (Table 6). This systematic review highlights the current state of knowledge among heterogeneous data and questions remaining to be investigated.

**Table 6:** Studies in progress or waiting for publication on Clinical Trial.

	**Titles**	**Applications**	**Identifications**
**Tobacco**			
1)	Developing a Smartphone App with Mindfulness Training for Teen Smoking Cessation	Craving to Quit(C2Q Teen app)	NCT02218281
2)	Automated Mobile Contingency Management for Smoking Cessation: a Pilot RCT	*Not specified*	NCT03739437
3)	Efficacy of a smoking cessation intervention using smartphones	SmokeFree Buddy	ISRCTN11154315
4)	Just Kwit: mobile Intervention for Tobacco Cessation	JustKwit	NCT03538678
5)	Smoking Cessation Smartphone App for Cancer Patients (Quit2Heal Study)	Quit2Heal	NCT03600038
6)	Impact of a smartphone application on smoking cessation: a randomized controlled trial	Stop-Tabac	ISRCTN11318024
7)	Study of effectiveness of a smartphone application for quitting smoking	BupaQuit	ISRCTN10548241
8)	Mindfulness Based Smoking Cessation Among Cancer Survivors	Craving to Quit	NCT04038255
**Alcohol**			
1)	The Efficacy of a Smartphone-based Support System to Reinforce Alcohol Abstinence in Treatment-seeking Patients	*Not specified*	NCT02385643
2)	The Effectiveness of a Smartphone Application in the Treatment of Alcohol Used Disorder	UControlDrink	NCT03396887
3)	Project Guard: reducing Alcohol Misuse/Abuse in the National Guard	SP-BI	NCT02860442
**Eating disorder**			
1)	Augmenting Specialty Eating Disorder Clinical Treatment With a Smartphone Application	Recovery record	NCT02484794
**Cannabis**			
1)	A randomized controlled trial of a smartphone application for people wanting to reduce or quit their use of cannabis	Joint Control	ACTRN12616000622404

## Conclusion

The current findings suggest that smartphone applications can effectively contribute to behavior change and craving management in SUDs and addictive disorders. However, to date, very few applications have been evaluated for validity. The evidence on the efficacy of mHealth addiction recovery applications are too limited at this time to be able to recommend them as an autonomous or complementary tool for the treatment of addictions. However, there is a signal that such applications could become significant contributors to treatment in the future. For that more rigorous RCTs including more homogeneous comparators are required, on larger scale and with longer-term evaluations so as to clarify the sustainability of the change in use behavior. Real-time interventions have immediate impact on behavior change. However, the long-term challenge is the prevention of relapse through the management of craving and global care. In that perspective, further research on mHealth is needed.

## Data Availability Statement

The original contributions presented in the study are included in the article/supplementary material, further inquiries can be directed to the corresponding author/s.

## Author Contributions

MA was the overall principal investigator of the study. RB and MA developed the review protocol and information search, and drafted the manuscript. RB, LF, and TG performed literature search, selected articles, and extracted information. J-MA and FS provided methodological support, critical revision, and editing of the manuscript. All authors significantly contributed to the manuscript and approved the final version.

## Conflict of Interest

The authors declare that the research was conducted in the absence of any commercial or financial relationships that could be construed as a potential conflict of interest.
